# Assessment of metal concentrations in oysters and shrimp from Atlantic Coast of the Democratic Republic of the Congo

**DOI:** 10.1016/j.heliyon.2019.e03049

**Published:** 2019-12-24

**Authors:** Robert B. Suami, Dhafer Mohammed M. Al Salah, César D. Kabala, J.-P. Otamonga, Crispin K. Mulaji, Pius T. Mpiana, John W. Poté

**Affiliations:** a University of Kinshasa (UNIKIN), Faculty of Science, Department of Chemistry, B.P. 190, Kinshasa XI, Democratic Republic of the Congo; b University of Kinshasa (UNIKIN), Faculty of Pharmaceutical Sciences, B.P. 212, Kinshasa XI, Democratic Republic of the Congo; c Department F.-A. Forel for Environmental and Aquatic Sciences and Institute of Environmental Sciences, School of Earth and Environmental Sciences, Faculty of Science, University of Geneva, Uni Carl Vogt, 66 Boulevard Carl-Vogt, Geneva 4, CH-1211, Switzerland; d King Abdulaziz City for Science and Technology, Joint Centers of Excellence Program, Prince Turki the 1st Street, Riyadh, 11442, Saudi Arabia; e Université Pédagogique Nationale (UPN). Croisement Route de Matadi et Avenue de la Libération. Quartier Binza/UPN, B.P. 8815, Kinshasa, République Démocratique du Congo

**Keywords:** Environmental science, Environmental pollution, Water pollution, Water quality, Environmental risk assessment, Heavy metal, Oysters, Shrimp, Bioaccumulation, Atlantic Ocean, Congo DR

## Abstract

Oysters and shrimp are abundant and commonly consumed seafood by the indigenous population of the Kongo central region of the Democratic Republic of the Congo (DRC). Literature reviews suggest that no data were available for the metal concentrations in these species. Consequently, the purpose of this study is to determine the metal concentrations in tissues of oysters (*Egeria congica*) and shrimp (*Macrobrachium* spp., *Parapenaeus* spp., *Penaeus* spp.) collected in November 2017 from the Atlantic Ocean Coast of DRC in the territory of Muanda. Metal levels in the seafood species studied here were put into context using international regulation for human consumption set by the Food and Agriculture Organization (FAO), Canadian Food Inspection Agency (CFIA), European Union (EU), and World Health Organization (WHO). Our results demonstrated that the concentration of heavy metals varied considerably between sampling sites and analyzed species (P < 0.05), with the values (in mg kg^1^) ranged between 0.05-0.41, 0.03–2.25, <LOD (limit of detection)-1.39, 4.19–60.46, 46.36–319.27, 0.18–3.74, 0.030.35, <LOD-0.01, 0.08–0.64, 1.12–25.76, 0.04–3.40 and 9.73–924.33 for Hg, Cr, Ni, Cu, Zn, Se, Cd, Sb, Pb, Mn, Co, and Fe, respectively. High concentrations of Cr, Mn, Co and Fe were found in *Egeria congica*; Cu in *Macrobrachium* spp., and Hg and Sb in *Parapenaeus* spp. Cu levels in 33.3% of *Macrobrachium* spp. and 16.7% *of Egeria congica* samples exceeded the FAO permissible limit of 30 mg kg^−1^ (wet weight (ww)). The concentration of Pb in one of six analyzed *Egeria congica* exceeded the EU permissible limit of 0.5 mg kg^−1^ (ww). The average values of Zn in all species exceeded the CFIA permissible limit of 50 mg kg^−1^ (ww). Metal pollution can be explained by several activities which include but not limited to oil exploitation, fuel traffic and tanker navigation, and erosion. High metal concentrations in investigated organisms present potential consumer human health risks.

## 1 Introduction

In marine environments, heavy metals contamination has been of great concern due to inherent toxicity of living organisms, potential ecological effects, vast sources, persistence, non-degradability, bioaccumulation, long biological half-lives, and public food safety (Besada et al., 2011; Wang et al., 2013; Le et al., 2016; Jonathan et al., 2017; Ross et al., 2017). Metal pollution is harmful to ecosystem biodiversity and can lead to the decline of sensitive native species or to a decrease in species abundance due to reproductive disorders and increased incidence of diseases (Wu et al., 2007; Kibria et al., 2012, 2016). Heavy metals can be bioaccumulated by marine organisms and even biomagnified through the food chain, resulting in elevated levels in predatory organisms (Scheifler et al., 2006; Rainbow and Luoma, 2011; Wang et al., 2013). The World Health Organization (WHO) recommends the control of heavy metals in food sources in order to assure public safety (Heidarieh et al., 2013; Olgunoğlu et al., 2015). The extent of heavy metal accumulation within an organism is affected by internal and external factors. Some internal factors include individual variability, body size and development stage, sex, breeding condition, brooding, molting and growth, behavior, storage, and excretion mechanisms. External factors include total amount of each metal bioavailability in the environmental medium, route of uptake, dissolved metals, dissolved oxygen, interactions between metals, sediment, food, seasonal effects and geographical differences (Chapman et al., 1996; Gokoglu et al., 2008; Chiarelli and Roccheri, 2014; Olgunoğlu et al., 2015).

Aquatic invertebrates such as mussels, oysters, shrimp or lobster, can be used to estimate the chemical and biological marine pollution (Rainbow, 2002; Dökmeci et al., 2014; Olgunoğlu et al., 2015). These species can exist in relatively polluted environments and integrate various indicators of water quality in their tissues (Dallinger and Rainbow, 1992; Rainbow, 2002; Chinnadurai et al., 2016). The accumulation of human pathogens within these species presents a public health issue and is being monitored worldwide especially for exported seafood (Food Code, 2013). Numerous studies have been conducted to assess the pollution status of marine ecosystems by heavy metals using different bioindicator organisms in order to evaluate the potential risks to human health (e.g. Lozano et al., 2010; Dionísio et al., 2013; Dökmeci et al., 2014; Nascimento et al., 2017; Welty et al., 2018). The use of these organisms, (like oysters and shrimp) as biological sentinels has proved to be useful for environmental monitoring due to their sensitivity and rapid response to environmental pollutants (Knakievicz, 2014; Szefer et al., 2006; Jonathan et al., 2017).

The concentrations of heavy metals in marine ecosystems, especially in seafood, deserve attention because of the potential ecological effects and the harmful effects on human health. Therefore, a better understanding of the current state of heavy metals pollution in coastal ecosystems is important for the seafood industry and public health concerns (Wang et al., 2005, 2013; Anandkumar et al., 2017). The Atlantic Coast of the DRC in the territory of Muanda is considered a geographical area of great importance because of the survival of a wide variety of plants, animals, and marine species including fishes, oysters, and shrimp (Mbomba, 2007). This coastal ecosystem is of great ecological interest and constitutes a major source of seafood production for local population consumption and export. However, anthropogenic activities reduce the productivity of this precious resource due to discharge of different varieties of pollutants, which include but not limited to oil spills and heavy metals from urban runoff and industrial wastewater (Mbomba, 2007; Suami et al., 2018). To our best knowledge, there are no previous studies assessing the levels of heavy metals in oysters and shrimp from the Atlantic Coast of the DRC.

The main objectives of this study were (i) to evaluate the concentration of metals including Hg, Cr,Ni, Cu, Zn, Se, Cd, Sb, Pb, Mn, Co, and Fe in oyster tissues (*Egeria congica*) and in three shrimp (*Macrobrachium* spp., *Parapenaeus* spp., *Penaeus* spp.) from the Atlantic Coast of the DRC in the territory of Muanda, (ii) to compare the metal concentrations in investigated seafood species with international regulations set for human consumption.

## 2 Materials and methods

### 2.1 Site description and seafood sampling

**Figure 1:**
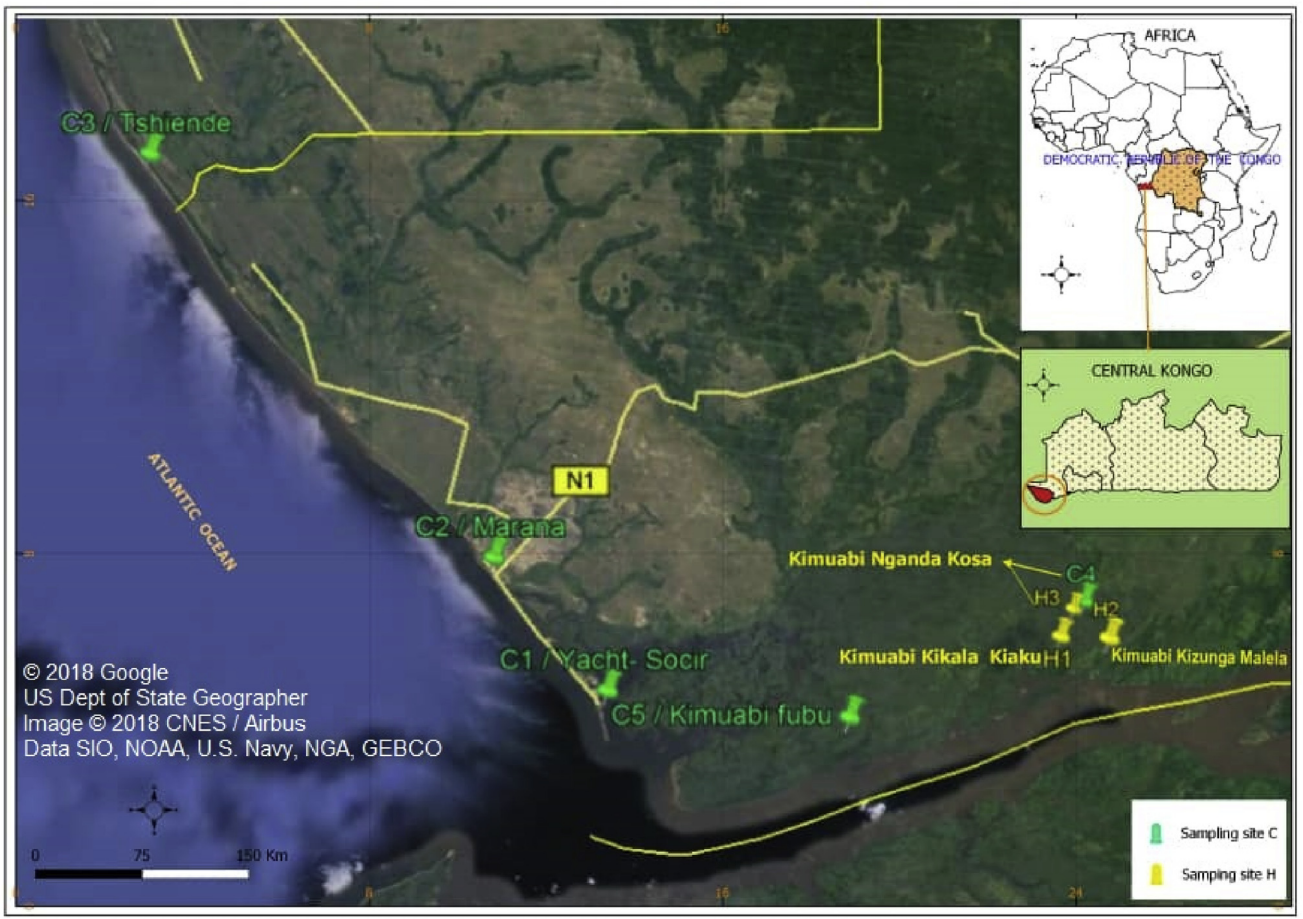
Map of the sampling location indicating: Location of Congo DR in Africa; Location of territory of Muanda in Congo DR; Atlantic Coast of Muanda in Central Congo Province, sampling stations (Adapted from Google Earth).

This research was performed in the Atlantic Coast of DRC in the territory of Muanda, located in the West of the Democratic Republic of the Congo, province of Kongo Central, district of Boma, Muanda territory (Figure 1). The institutional authority in the DRC approved the field studies and sampling.

**Figure 2:**
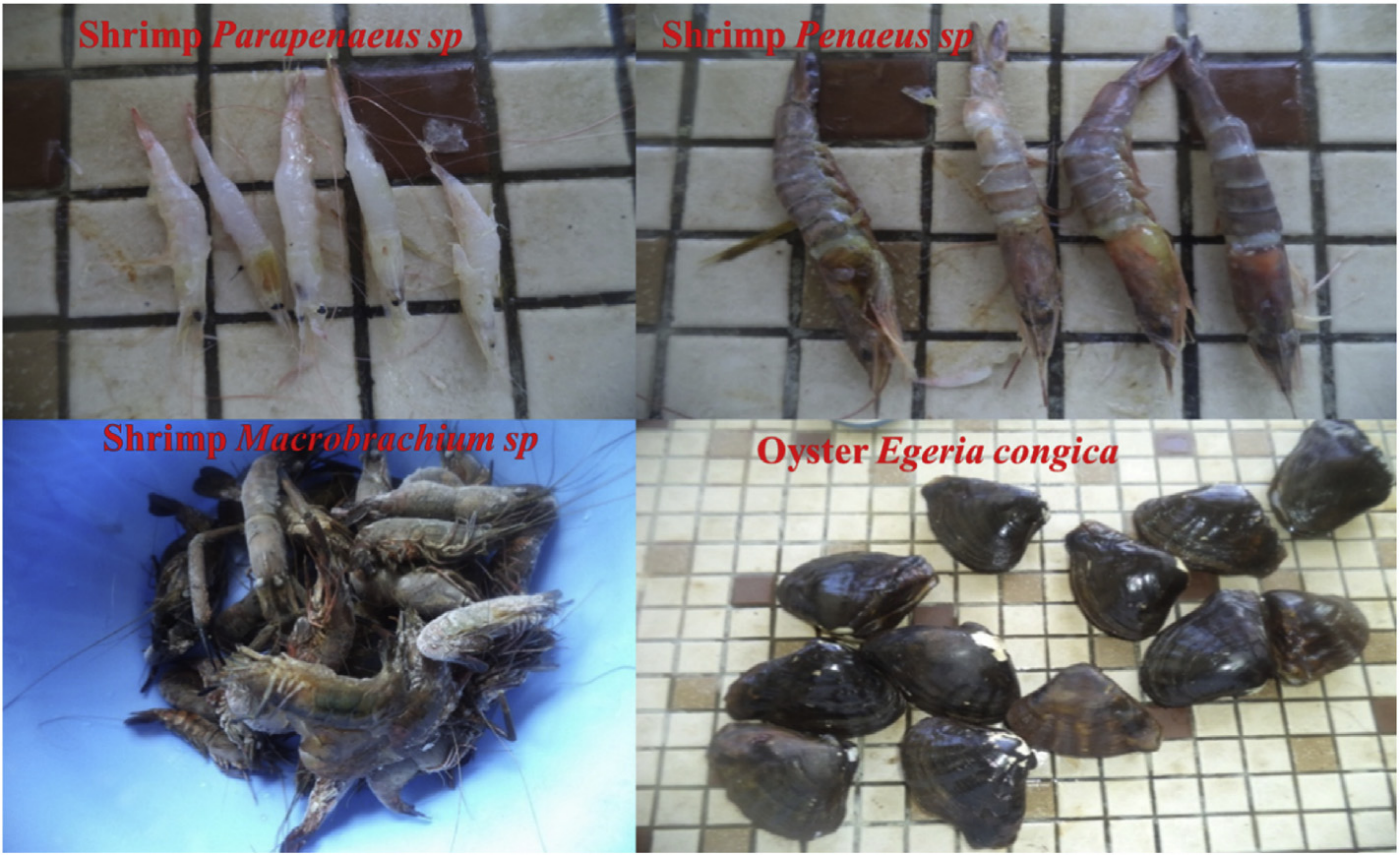
Analyzed oysters and shrimp from Atlantic Coast of Muanda, Democratic Republic of the Congo. Photos taken by Robert B. Suami in November 2017.

Oysters (*Egeria congica* (n = 6)) and shrimp; (*Macrobrachium* spp. (n = 4), *Parapenaeus* spp. (n = 2) and *Penaeus* spp. (n = 3)) were collected in November 2017 (Figure 2). The three sites for oysters sampling are named and labelled; Kimuabi Kikala Kiaku (H1), Kimuabi Kizunga Malela (H2) and Kimuabi Nganda Kosa (H3), two samples per site. The five sites for shrimp sampling are named and labelled; Yacht-Socir (C1), Marana (C2), Tshiende (C3), Kimuabi Nganda Kosa (C4) and Kimuabi Fubu (C5), two samples per site except for the site C1 (one sample). The studied organisms were collected using different local fishing nets. These sampling sites were selected according to the suggestion performed in our previous study (Suami et al., 2018).

All samples were stored in an icebox at 4 °C that were transported to the laboratory for different treatments within 24 h. After preliminary treatments, the samples were sent to the Department F.-A. Forel, the University of Geneva for analysis.

### 2.2 Oysters and shrimp samples preparation

The collected samples were washed with Milli-Q water, length measured using a ruler, dissected, and the tissues were separated on-site, frozen, and stored at -20 °C in clean polypropylene bottles until acid digestion. The samples were weighted, lyophilized (Adolf Kühner, Birsfelden, Switzerland), and water content was calculated as performed in our previous study (Ngelinkoto et al., 2014). The digestion of samples was performed according to the methods described by Rashed (2001), Sivaperumal et al. (2007) and Ngelinkoto et al. (2014) with minor modifications. Briefly, a portion of edible tissue from each sample was freeze-dried (Adolf Kühner, Birsfelden, Switzerland) and ground to obtain a fine powder. Approximately 1 g was digested in 10 mL of a suprapur HNO_3_ (Nitric acid 65% Suprapur, Merck KGaA, Darmstadt Germany)-HClO_4_ (Perchloric acid 70%, Merck KGaA, Darmstadt Germany) mixture (3:1), in Teflon bombs and heated overnight at 110 °C. The digested samples were cooled at room temperature and centrifuged. Inductively coupled plasma mass spectroscopy (ICP-MS) analysis was performed after dilution with suprapur 1% HNO_3_ (Nitric acid 65% Suprapur, Merck KGaA, Darmstadt Germany). Acid dilutions were performed using ultrapure water (Millipore, Milli-Q, 18MW, Merck, Darmstadt, Germany).

### 2.3 Metal analysis in oysters and shrimp samples by ICP-MS

The concentrations of metals Cr, Ni, Cu, Zn, Se, Cd, Sb, Pb, Mn, Co, and Fe in digested oysters and shrimp samples were measured using ICP-MS (model 7700 series, Agilent, Santa Clara, CA, USA). A collision/reaction cell (helium mode) and interference equations are utilized to correct for spectral interferences. The standard solutions at different concentrations (0, 0.2, 1, 5, 10, 20, 50 and 100 μg L^−1^) prepared from ICP multi-element standard solution Merck IV, 1000 mg L^−1^, Merck KGaA, Darmstadt Germany) and other mono element solutions (Se and Sb, Merck KGaA, Darmstadt Germany) for ICP-MS analysis, were used for calibration. The limit of detection (LOD) was calculated as 3 times standard deviation of the blanks and was less than 0.001 μg L^−1^ for all analyzed elements. The results are expressed in mg kg^−1^ wet weight (ww) and calculated with average values of water content in oysters and shrimp tissues as described previously (Garcia-Bravo et al., 2010).

### 2.4 Mercury analysis in oysters and shrimp samples

The total mercury (THg) analysis in oysters and shrimp samples was carried out using atomic absorption spectrophotometry (AAS, Advanced Mercury Analyzer; AMA 254, Altecs.r.l., Czech Rep.) as described by Garcia-Bravo et al. (2011). The method is based on sample combustion, gold amalgamation, and AAS. The results are expressed in mg kg^−1^ wet weight (ww).

### 2.5 Quality control and statistical analysis

For all analyses, triplicate measurements have been performed on oysters and shrimp samples. The certified reference material DORM-3 (National Research Council, Ottawa, Ontario Canada) was used to verify the quality, the precision and the reliability of the results. DORM-3 was prepared and analyzed in the same manner as the oysters and shrimp samples. Statistical treatment of data (ANOVA followed to Bartlett's Test and in case of non-applicability, Kruskal-Wallis test**)** has been realized using Epi Info version 3.5.4 in order to compare the averages concentrations between species studied. The Pearson correlation was performed using SPSS Statistics version 20 in order to measure the degree of correlation between species length and heavy metal concentration in the samples and to have information on their probable sources and pathways (Manta et al., 2002; Al-Khashman and Shawabkeh, 2006). The significance of fixed effects was assessed by a t-test using a significance level of 5%.

## 3 Results and discussion

### 3.1 Quality control and certified reference material values of metal concentrations

For ICP-MS analysis, the total variation coefficients of triplicate sample measurements were below 3% and chemical blanks for the procedure were less than 2% of the sample signals. For AMA, the detection limit (3 SD blank value) was 0.005 mg kg^−1^ and the triplicates didn't vary more than 2%. The obtained values of analyzed metals by ICP-MS for the reference material DORM-3 were in the certified range. The results are reported in Table 1. The recovery values ranged from 92.6 to 98.6% for ICP-MS analysis and 97.4% for Hg analysis by AMA.

**Table 1:** Recovery values of certified reference material DORM-3 (in mg kg^−1^)∗.

Element	Certified value	Measured value (n = 3)	Recovery (%)
Cr	1.89 ± 0.17	1.75 ± 0.30	92.59
Ni	1.28 ± 0.24	1.26 ± 0.63	98.43
Cu	15.5 ± 0.63	14.7 ± 1.22	94.83
Zn	51.3 ± 3.1	49.80 ± 2.70	97.07
Se	1.40 ± 0.09	1.38 ± 0.06	98.57
Cd	0.29 ± 0.02	0.27 ± 0.00	93.10
Pb	0.39 ± 0.05	0.38 ± 0.00	97.43
Fe	395 ± 50	377 ± 27.0	95.44
Hg	0.38 ± 0.06	0.37 ± 0.01	97.36

∗ The recovery values from the ICP-MS and AMA for Hg. Triplicate measurements for reference material (DORM-3) were in good agreement with the provided certified values and above 92.5% for all elements.

### 3.2 Concentration of metals in muscles of oysters and shrimp samples

The range and mean of length for oysters and shrimp species are presented in Table 2. The concentrations of Hg, Cr, Ni, Cu, Zn, Se, Cd, Sb, Pb, Mn, Co, and Fe in tissues of *Macrobrachium* spp., *Parapenaeus* spp., *Penaeus* spp. and *Egeria congica* varied considerably between species (P˂0.05) (Table 3). The average concentrations of Fe, Zn and Cu are considerably higher when compared with other metals. In general, the order of average concentrations of analyzed metals was different amongst species and in the following order (Table 3): Zn>Cu>Fe>Mn>Se>Cd >Pb>Hg>Ni>Co >Cr>Sb in *Macrobrachium* spp., Fe>Zn>Cu>Mn>Se>Hg>Cd>Cr=Pb>Ni=Co>Sb in *Parapenaeus* spp., Fe>Zn>Cu>Se>Mn>Pb>Hg>Cr>Cd>Co>Sb>Ni in *Penaeus* spp. and Fe>Zn>Mn>Cu>Co>Se>Cr>Ni>Pb>Hg>Cd>Sb in *Egeria congica*.

**Table 2:** Range and mean of length of oysters and shrimp species.

Species	Length (cm)	
	Range	Mean ± SD
**Shrimp species**		
*Macrobrachium* spp. (n = 4)	6.20–15.00	9.38 ± 2.85
*Parapenaeus* spp. (n = 2)	6.17–13.81	9.67 ± 3.42
*Penaeus* spp. (n = 3)	6.30–11.10	8.98 ± 1.40
**Oysters species**		
*Egeria congica* (n = 6)	3.80–9.40	5.54 ± 1.54

**Table 3:** Concentrations of metals (mg kg^−1^) from analyzed oysters species and shrimp from Muanda Coast^a^.

Species	Metal concentrations in mg kg^−1^ ww											
	THg		Cr		Ni		Cu		Zn		Se	
	Range	Mean ± SD	Range	Mean ± SD	Range	Mean ± SD	Range	Mean ± SD	Range	Mean ± SD	Range	Mean ± SD
**Shrimp species**												
*Macrobrachium* spp. (n = 4)	0.05–0.11	0.07 ± 0.03	0.03–0.07	0.04 ± 0.02	<LD-0.23	0.06 ± 0.12	27.71–60.46	41.84 ± 13.66	53.33–67.79	59.41 ± 6.10	0.31–1.01	0.59 ± 0.33
*Parapenaeus* spp. (n = 2)	0.26–0.41	0.34 ± 0.11	0.04–0.34	0.19 ± 0.21	<LD-0.11	0.06 ± 0.08	16.66–24.16	20.41 ± 5.30	47.07–84.84	65.95 ± 26.71	1.22–1.62	1.42 ± 0.28
*Penaeus* spp. (n = 3)	0.05–0.25	0.17 ± 0.10	0.08–0.10	0.09 ± 0.01	<LD	<LD	16.01–18.71	17.68 ± 1.45	55.90–85.44	69.28 ± 14.96	1.17–3.74	2.54 ± 1.30
**Oysters species**												
*Egeria Congica* (n = 6)	0.20–0.37	0.30 ± 0.07	0.12–2.25	1.06 ± 0.79	0.00–1.39	0.49 ± 0.48	4.19–37.16	16.28 ± 12.07	46.36–319.27	112.65 ± 106.04	0.18–3.54	1.89 ± 1.38
Permissible level (mg.kg^−1^ wet wt)	1^b^		12^c^		70^d^		30^e^		50^f^			

Species	Metal concentrations in mg kg^−1^ ww											
	Cd		Sb		Pb		Mn		Co		Fe	
	Range	Mean ± SD	Range	Mean ± SD	Range	Mean ± SD	Range	Mean ± SD	Range	Mean ± SD	Range	Mean ± SD
**Shrimp species**												
*Macrobrachium* spp.(n = 4)	0.12–0.26	0.21 ± 0.07	<LD	<LD	0.14–0.18	0.16 ± 0.02	3.57–5.40	4.24 ± 0.85	0.04–0.07	0.05 ± 0.02	9.73–19.42	14.26 ± 4.00
*Parapenaeus* spp. (n = 2*)*	0.13–0.35	0.24 ± 0.16	0.01–0.01	0.01 ± 0.00	0.12–0.25	0.19 ± 0.09	1.87–4.80	3.34 ± 2.07	0.04–0.08	0.06 ± 0.03	68.12–121.68	94.90 ± 37.87
*Penaeus* spp. (n = 3)	0.05–0.06	0.06 ± 0.01	<LD-0.01	0.01 ± 0.00	0.18–0.24	0.21 ± 0.03	1.12–1.91	1.60 ± 0.42	0.04–0.06	0.05 ± 0.01	49.96–125.45	78.87 ± 40.73
**Oysters species**												
*Egeria Congica* (n = 6*)*	0.03–0.31	0.14 ± 0.11	0.00–0.01	0.00 ± 0.00	0.08–0.64	0.34 ± 0.21	1.87–25.76	17.46 ± 9.65	0.52–3.40	1.91 ± 1.14	45.22–924.33	457.30 ± 324.81
Permissible level (mg.kg ^−1^ wet wt)	1^b^				0.5^g^							

SD: Standard deviation.

a Total variation coefficients for triplicate measurements are smaller than 2% for ICP-MS analysis.

b EC, 2001.

c US FDA, 1993a.

d US FDA, 1993b.

e FAO, 1983.

f CFIA, 2011.

g EU, 2008.

The concentration of Hg in the tissues of oysters and shrimp was within the permissible levels for human consumption set by Food and Agriculture Organization (FAO), European Union (EU), and World Health Organization (WHO). The values ranged between 0.20 -0.37 and 0.05–0.41 mg kg^−1^ wet weight (ww), for oysters and shrimp respectively. The maximum value of 0.41 mg kg^−1^ was observed in *Parapenaeus* spp. and this concentration is higher compared to that observed by Dökmeci et al. (2014) in *Parapenaeus longirostris* from the Marmara Sea Coast in Tekirdağ (0.18 mg kg^−1^). Hg is toxic to humans and is not an essential element for aquatic living organisms and more attention have to be considered even at low concentration, because of its rapid biomagnification in fishes and other seafood (Hobson and Welch, 1992; Dehn et al., 2006; Niane et al., 2015). The effects of Hg on human health are closely related to its form of existence, and the toxicity of methyl mercury (MeHg) is higher than that of inorganic mercury. It has been demonstrated that 70–97% of Hg in fish muscle and shellfish tissues are in the form of MeHg (Bloom, 1992; Garcia-Bravo et al., 2010). Methyl mercury is the main stable organic form of Hg that is absorbed by the human body through the consumption of fish and seafood and is well known as neurotoxicant (Garcia-Bravo et al., 2010, 2011; Guynup and Safina, 2012; Niane et al., 2015; Bosch et al., 2016; Salgado-Ramírez et al., 2017).

The concentration of Cr in oyster and shrimp samples ranged from 0.12 to 2.25 mg kg^−1^ and 0.03–0.34 mg kg^−1^ respectively. A significant difference (p < 0.05) was observed in the bioaccumulation of Cr between oysters and shrimp samples (Table 3). The average concentrations of Cr in the oysters and shrimp indicated that the oysters had accumulated more Cr as compared with the shrimp. The maximum concentration of Cr (2.25 mg kg^−1^) was recorded in oysters (*Egeria congica*). However, this value is below the permissible limit (12 mg kg^−1^) set by Food and Drug Administration Guidance Document for Chromium in Shellfish (US FDA, 1993a), lower than 23 mg kg-1as reported by Hernandez et al. (2003) for oysters *Crassostrea virginica* from Bahia Cienfuegos of Cuba, and also lower than 104 mg kg-1for oysters *Crassostrea gigas* from SW Gulf of California Coast, Mexico (Jonathan et al., 2017). As for in humans and animals, Cr (III) is an essential and nutritious element that plays an important role in the metabolism of glucose, lipids and proteins by facilitating the interaction of insulin with its receptor site (Anderson, 1997). It is considered less toxic, although at high doses it may inhibit certain enzyme systems or react with organic molecules. Cr (VI), whose dietary intake and domestic emissions are major sources of exposure in the general population, is a potent oxidant causing cellular damage (Merzenich et al., 2001; Costa and Klein, 2006). It is allergenic and considered a carcinogen by inhalation (lungs) for humans (IARC, 1990).

The concentrations of Ni varied between <LD to 1.39 mg kg^−1^ in oysters and between <LD to 0.23 mg kg^−1^ in shrimp. There was no significant difference (p > 0.05) in the bioaccumulation of Ni between oysters and shrimp. The values of Ni in the shrimp samples obtained from the Northern Inner Shelf of the Sea of Marmara ranged from 1.4 to 7.5 mg kg^−1^ (Kurun et al., 2007), so they are higher than those found in shrimp in this study. The maximum Ni concentration observed in oysters (1.39 mg kg^−1^) was lower than the maximum value of 42.33 mg kg^−1^ obtained by Jonathan et al. (2017).The Ni levels detected in all the samples were below the allowable limit of 70 mg kg^−1^ (US FDA, 1993b). Food is the main source of Ni exposure in the general population (Kristiansen et al., 1997; Sharma, 2007).

Copper contents in investigated samples ranged from 4.19 to 37.16 mg kg^−1^ and from 16.01 to 60.46 mg kg^−1^ for oysters and shrimp, respectively. There was a significant difference (p < 0.05) between average Cu concentration in all species. The highest concentration (60.46 mg kg^−1^) of Cu was recorded in the shrimp species *Macrobrachium* spp. and was higher than that found by Idrus et al. (2018) in the species *Macrobrachium rosenbergii* (below than 30 mg kg^−1^). The maximum concentration (37.16 mg kg^−1^) in oysters was lower than the values of 67 mg kg^−1^observed by Paez-Osuna et al. (1991), 76.5 mg Kg^−1^ by Frías-Espericueta et al. (2005) in *Crassostrea corteziensis* along the Mexican Pacific Coast and 63.37 mg kg^−1^ by Jonathan et al. (2017) in *Crassostrea gigas* from SW Gulf of California Coast, Mexico. On other hand, this concentration was higher than that found by Sivaperumal et al. (2007) (24.1 mg kg^−1^). About 16.7% of analyzed oyster samples and 33.3% of shrimp (*Macrobrachium* spp.) samples exceeded the FAO permissible limit of 30 mg kg^−1^ (wet weight). Cu is an essential trace mineral that plays major roles in the immune, hematopoietic, cardiovascular, nervous systems, bone and blood vessel, cholesterol regulation, and oxidative stress control (Schümann et al., 2002; Underwood and Suttle, 1999). However, if excessively accumulated, Cu can be toxic and it may be harmful in higher doses, causing gastrointestinal distress, damage to liver, the immune system, neurological system and reproductive ability (Schümann et al., 2002; ATSDR, 2007).

In oysters, the concentrations of Zn ranged from 46.36 to 319.27 mg kg^−1^ while those in shrimp ranged from 47.07 to 85.44 mg kg^−1^. There was no significant difference (p > 0.05) between the average Zn concentrations in the different studied species. The average values of Zn in all species exceeded the CFIA permissible limit of 50 mg kg^−1^. The maximum value of 319.27 mg kg^−1^ was observed in oyster (*Egeria congica)*. The recorded values of Zn in oysters in this study was higher than that found by Sivaperumal et al. (2007) (165.00 mg kg^−1^) and lower than that recorded by Jonathan et al. (2017) (416.67 mg kg^−1^). Zinc is an essential metal in small quantities for the life of a large number of organisms and in particular for aquatic organisms. As a result, its potential biomagnification is low especially in marine environments (Hunt and Hedgecott, 1992), similar to our studied area. Zn is essential for many metabolic and enzymatic functions in humans such as growth and development, testicular maturation, neurological function, wound healing and immune competence (Calabrese et al., 1985). It has several roles in the hormonal and biochemical functions of various endocrine organs (Walsh et al., 1994; ATSDR, 2012). Zn is one of the metals considered less toxic to humans, and deficiency problems are more common and more severe than those of toxicity (Martin, 1996). Deficiency of this metal can cause stunting, loss of taste and possible decrease infertility. Toxicity with high levels of Zn is rare. This metal may have a protective effect against the toxicities of Cd, and Pb (Calabrese et al., 1985). Nevertheless, it may exert some toxicity at higher doses.

In oysters, the concentrations (in mg kg^−1^) of Se, Cd and Pb ranged from 0.18 to 3.54, 0.03 to 0.31 and 0.08–0.64 mg kg^−1^, respectively, and in shrimp from 0.31 to 3.74, 0.05 to 0.35and 0.12–0.25 mg kg^−1^, respectively (Table 3). It may be pointed out that the absorption difference of Se, Cd and Pb by the shrimp and the oysters was not statistically significant (p > 0.05). Cd concentrations were lower than the allowable limit of 1 mg kg^−1^ (EC, 2001). Cadmium is highly toxic to humans and has a long biological half-life which is suitable for bioaccumulation (Erasmus et al., 2004; EFSA, 2009). Several studies conducted on groups of individuals who have been chronically contaminated with cadmium, reveal the occurrence of kidney disease (Diamond and Zalups, 1998; Nordberg et al., 1975; Silva et al., 2004), pulmonary involvement (Nordberg et al., 1975) and bone diseases (Silva et al., 2004; Nordberg et al., 1975; Alfvén et al., 2000). The maximum Pb concentration of 0.64 mg kg^−1^ was recorded in the oysters samples and was lower than the values ranged from 1.8 to14 mg kg^−1^ in pearl oysters *Pictada radiate* from Bahrain (Al-Sayed et al., 1994) and of 4.67 mg kg^−1^ in *Crassostrea gigas* from SW Gulf of California Coast, Mexico (Jonathan et al., 2017). The Pb maximum concentration of 0.25 mg kg^−1^ in shrimp samples was found in *Parapenaeus* spp. and was lower than the value of 2.12 mg kg^−1^ in *Parapenaeus longirostris* from the Marmara Sea Coast in Tekirdağ recorded by Dökmeci et al. (2014). The Pb concentration in one of six analyzed oysters samples exceeded the EU (EU, 2008) permissible limit of 0.5 mg kg^−1^ (wet weight). This sample also contained the highest value of Cu. A high concentration of Pb from food can cause neurological problems, hematological effects, kidney failure, hypertension and cancer (Goyer and Clarkson, 2001; Muñoz-Olivas and Camara, 2001; Rubio et al., 2005). Sb concentrations in oysters and shrimp samples were negligible with maximum value of 0.01 mg kg^−1^.

The concentration of Mn in oysters samples ranged from 1.87 to 25.76 mg kg^−1^ while in shrimp ranged from 1.12 to 5.40 mg kg^−1^. There was a significant difference (p < 0.05) between the averages concentrations measured in different species. The maximum concentration of Mn (25.76 mg kg^−1^) observed in the oysters' samples in this study was higher than that found by Sivaperumal et al. (2007) (3.75 mg kg^−1^) and by Ragi et al. (2017) (8.50 mg kg^−1^). The lowest concentration (1.12 mg kg^−1^) was recorded in *Penaeus* spp. Mn is an essential and ubiquitous element in food, environment, and biota. Its effects in different concentrations are well documented and detailed (e.g. Torrente et al., 2005; Wasserman et al., 2006; ATSDR, 2012).

The Co concentrations determined in this study varied between 0.52 to 3.40 mg kg^−1^ in the oysters and between 0.04 to 0.08 mg kg^−1^ in the shrimp (Table 3). A comparison of averages Co concentrations in the oysters and shrimp indicated that there was a significant difference between species (p < 0.05). The highest concentration of Co (3.40 mg kg^−1^) observed in the oyster species in the current study was higher than that found by Sivaperumal et al. (2007) (0.85 mg kg^−1^). Co is an essential element and it is also used as a food additive in ruminants to allow the production of vitamin B12 by their digestive flora. Nevertheless, it has been classified as a “possible carcinogen” by the International Agency for Research on Cancer (INERIS, 2006).

The concentrations of Fe in oysters’ tissues ranged from 45.22 to 924.33 mg kg^−1^ while those in tissues of shrimp ranged from 9.73 to 125.45 mg kg^−1^. There was a significant difference (p < 0.05) between the averages Fe concentrations measured in different species. Most of the oysters samples (five of six) had higher iron concentrations than the shrimp samples. The highest concentration of 924.33 mg kg^−1^ was found in *Egeria congica* while the lowest concentration of 9.73 mg kg^−1^ was recorded in *Macrobrachium* spp. However, there are currently no national nor international limits to the human consumption of Fe. According to several parameters, Fe can catalyze many reactions and in some cases produce toxic effects in the body (Isidori et al., 2018; Bechaux et al., 2018).

In general, the results of this study showed that oysters accumulated more Cr, Mn, Co and Fe than shrimp species while shrimp, especially *Macrobrachium spp*, which accumulated more Cu than oysters and other shrimp species. Hg and Sb are more accumulated in *Parapenaeus* spp*.* then in other species. Significant inter-species differences were therefore founded in this study among heavy metal levels in the groups of marine organisms from Atlantic Coast of Muanda, Democratic Republic of the Congo. Several possible explanations could account for the variation in metal accumulation rate. Metabolic rate of the organisms, exposure route, metal mobility, bioavailability and species of the chelator present in water and sediment of the coastal areas and time spent in the contaminated water are among the major factors (Gokoglu et al., 2008; Offem and Ayotunde, 2008; Vinodhini and Narayanan, 2008; Gundogdu et al., 2011). In addition, factors such as pH, temperature, salinity, nutrients, organic matter, organic carbon and environmental conditions of the ecosystem influence the bioavailability and bioaccumulation rate of metals (Kamala-Kannan and Krishnamoorthy, 2006). In this study, there was no significant difference in the bioaccumulation of Ni, Cd, Se, Pb, and Zn between oysters and shrimp. In general, oysters present higher concentrations of heavy metals than shrimp as reported also by Wang et al. (2013). The two metals that exceed the ceiling limits of concentrations dictated by international organizations are Zn and Cu and are the highest among metals in all species as reported in other studies (Suami et al., 2018). The high concentrations of Zn and Cu in the tissues of the studied species could be attributed to high concentration of these metals in the surrounding environment whether the source could be the erosion of crustal metals (to be explored in depth) or wastewater discharge. On the other hand, Sb was the least accumulated metal in almost all samples. The order of variation of metal concentrations in the oyster samples from different sites of this study is almost similar to that obtained by Jonathan et al. (2017) in farmed pacific oysters *Crassostrea gigas* from SW Gulf of California at Mexico.

### 3.3 Statistical correlations

Pearson's correlation coefficients were calculated for oysters and shrimp (*Macrobrachium* spp*.* and *Penaeus* spp.) and presented in Tables 4 and 5.

**Table 4:** Pearson correlation coefficients of selected parameters^c^ analyzed in oysters species.

	Length	Hg	Cr	Ni	Cu	Zn	Se	Cd	Sb	Pb	Mn	Co	Fe
Length	1.000	0.718	0.774	0.275	0.011	-0.235	0.435	0.151	**0.815**^a^	0.174	0.398	0.286	0.764
Hg		1.000	0.776	-0.302	-0.107	-0.267	0.436	0.137	0.543	0.258	0.620	0.315	0.811
Cr			1.000	-0.340	0.494	-0.300	**0.873**^a^	0.671	0.743	0.721	**0.871**^a^	0.789	0.997
Ni				1.000	-0.470	-0.538	-0.553	-0.569	0.024	-0.630	-0.687	-0.599	-0.362
Cu					1.000	**0.957**^b^	**0.843**^a^	**0.958**^b^	0.247	**0.908**^a^	0.682	**0.908**^a^	0.458
Zn						1.000	0.711	0.907	**0.079**^a^	**0.844**^b^	0.544	0.813	0.266
Se							1.000	**0.931**^a^	0.583	**0.937**^a^	0.929	0.983	**0.853**^a^
Cd								1.000	0.363	**0.979**^b^	**0.809**^a^	0.978	0.646
Sb									1.000	0.294	**0.422**^a^	0.444	0.704
Pb										1.000	0.886	**0.983**^a^	0.710
Mn											1.000	0.908	**0.879**^a^
Co												1.000	0.771
Fe													1.000

a Correlation is significant at the 0.05 level (2-tailed).

b Correlation is significant at the 0.01 level (2-tailed).

c Parameters include length and metals (n = 6, statistically significant coefficients (P < 0.05; P < 0.01) are in bold.

**Table 5:** Pearson correlation coefficients of selected parameters^c^ analyzed in shrimp species (*Macrobrachium* spp. and *Penaeus* spp.).

	Length	Hg	Cr	Ni	Cu	Zn	Se	Cd	Sb	Pb	Mn	Co	Fe
Length	1.000	-0.190	-0.168	-0.312	0.404	0.051	-0.296	-0.081	-0.305	-0.346	0.022	-0.705	-0. 385
Hg		1.000	**0.768**^a^	-0.179	-0.649	-0.338	**0.879**^b^	-0.641	**0.946**^b^	0.002	-0.497	-0.191	**0.845**^a^
Cr			1.000	-0.211	**-0.919**^b^	0.130	0.701	**-0.895**^b^	0.689	0.343	**-0.826**^a^	-0.270	**0.831**^a^
Ni				1.000	0.187	-0.413	-0.363	0.523	-0.258	-0.488	0.352	0.674	-0.237
Cu					1.000	-0.154	-0.567	**0.775**^a^	-0.606	-0.427	0.699	0.001	-0.711
Zn						1.000	-0.001	-0.431	-0.152	**0.865**^a^	-0.600	-0.153	-0.006
Se							1.000	-0.694	**0.944**^b^	0.375	-0.639	-0.251	**0.933**^b^
Cd								1.000	-0.645	-0.548	**0.941**^b^	0.477	-0.718
Sb									1.000	0.224	-0.557	-0.080	**0.838**^a^
Pb										1.000	-0.676	-0.034	0.369
Mn											1.000	0.323	-0.674
Co												1.000	-0.249
Fe													1.000

a Correlation is significant at the 0.05 level (2-tailed).

b Correlation is significant at the 0.01 level (2-tailed).

c Parameters include length and metals (n = 7, statistically significant coefficients (P < 0.05; P < 0.01) are in bold.

Pearson correlation coefficients of selected parameters analyzed in oyster species are presented in Table 4. A significant positive correlation was observed between the length and Sb (r = 0.815; p < 0.05). Furthermore, copper showed a strong positive correlation with Se (r = 0.843, p < 0.05), Pb (r = 0.908, p < 0.05), Co (r = 0.908; p < 0.05), Cd (r = 0.958; p < 0.01) and Zn (0.957; p < 0.01). Selenium showed a significant positive correlation with Cd, Pb, Fe and Cr (r = 0.931; r = 0.937; r = 0.853; r = 0.873; p < 0.05 respectively). Cr, Cd, Sb and Fe presented a significant positive correlation with Mn (r = 0.871; r = 0.809; r = 0.422; r = 0.879; p < 0.05). Lead showed a significant positive correlation with Zn (r = 0.844; p < 0.01), Cd (r = 0.979; p < 0.01) and Co (r = 0.983; p < 0.05). There was also a very low positive correlation between Zn and Sb (r = 0.079; p < 0.05). The results showed a significant positive correlation between the metals Cr, Se, Mn, Cu, Pb, Co, Zn, Cd, Sb and Fe, but Hg and Ni did not show significant correlations with these metals (Table 4). Therefore, these metals (Cr, Se, Mn, Cu, Pb, Co, Zn, Cd, Sb and Fe) could probably have common sources (Suami et al., 2018), which may be industrial sources and urban discharge into streams and rivers upstream of the sampling sites. On the other hand, given the high concentration of crustal metals present such Mn and Fe, and the proximity of a nearby studied area, erosion can be a potential major source of metal discharges into the studied marine environment.

Pearson correlation coefficients of selected parameters analyzed in shrimp species are presented in Table 5. No significant correlation was observed between length and concentration of heavy metals. Hg showed a significant positive correlation with Cr (r = 0.768; p < 0.05), Fe (r = 0.845; p < 0.05), Se (r = 0.879; p < 0.01) and Sb (r = 0.946; p < 0.01). Fe presented a significant positive correlation with Cr (r = 0.831, p < 0.05), Sb (r = 0.838; p < 0.05) and Se (r = 0.933; p < 0.01). Cd showed a strong positive correlation with Cu (r = 0.775; p < 0.05) and Mn (r = 0.941; p < 0.01). A significant and positive correlation was also observed between Zn and Pb (r = 0.865; p < 0.05) and between Se and Sb (r = 0.944; p < 0.01). The strong positive correlation between heavy metals may reflect similar levels of contamination and/or releases from the same sources of pollution (Håkanson and Jansson, 1983; Li et al., 2009). These results showed that the same factors (as explained above) were effective in the accumulation of these metals (Kurun et al., 2007). The significant positive correlations among these metals in the tissues of shrimp may reflect a common source and similar accumulation behavior and interactions in the Atlantic Coast of Muanda (Suami et al., 2018; Anandkumar et al., 2017). On the other hand, Cr showed a significant negative correlation with Mn (r = -0.826; p < 0.05), Cu (r = -0.919; p < 0.01) and Cd (r = -0.895; p < 0.01). The significant negative correlation between metals showed that different mechanisms play role in the accumulations of these metals (Kurun et al., 2007).

## 4 Conclusion

This study provides the first measurement of metals data in four important seafood species (oysters (*Egeria congica*) and shrimp (*Macrobrachium* spp., *Parapenaeus* spp., *Penaeus* spp.)) from Atlantic Coast of Muanda. The obtained results demonstrate the significant difference in the accumulation of metals Hg, Cr, Cu, Sb, Mn, Co and Fe between oysters and shrimp species and no significant difference for Ni, Zn, Se, Cd and Pb. In all analyzed samples (both of oysters and shrimp species), the metal concentrations were below levels of concern for seafood safety, except in the case of Cu and Pb levels in some samples of *Macrobrachium* spp., and *Egeria Congica* spp. The data from this study as well as from our previous research performed in this area (Suami et al., 2018), propose a continuous evaluation/monitoring program for metal pollution and their accumulation in biota for potential changes. Additionally, several activities in the Atlantic Coast of Muanda, including oil exploitation, fuel traffic and tanker navigation, and input effluent waters from the Congo River, the assessment of toxic metals, in water, sediment, and marine organisms is necessary and highly recommended to evaluate any further environmental deterioration. Finally, further studies with more organism samples from the studied area are recommended to fully evaluate consumer human health risks.

## Declarations

### Author contribution statement

Robert B. Suami, Dhafer Mohammed M. Al Salah: Performed the experiments; Analyzed and interpreted the data; Contributed reagents, materials, analysis tools or data; Wrote the paper.

César D. Kabala, J-P. Otamonga, Pius T. Mpiana: Conceived and designed the experiments; Wrote the paper.

Crispin K. Mulaji, John Poté: Conceived and designed the experiments; Analyzed and interpreted the data; Contributed reagents, materials, analysis tools or data; Wrote the paper.

### Funding statement

This work was supported by the Swiss National Science Foundation (grant no. 31003A_173281/1) and the Academy of Research and Higher Education of Wallonia- Brussels (ARES).

### Competing interest statement

The authors declare no conflict of interest.

### Additional information

No additional information is available for this paper.
